# Comparative study of the quality indices, antioxidant substances, and mineral elements in different forms of cabbage

**DOI:** 10.1186/s12870-024-04857-4

**Published:** 2024-03-14

**Authors:** Zhibin Yue, Guobin Zhang, Jie Wang, Jue Wang, Shilei Luo, Bo Zhang, Zhaozhuang Li, Zeci Liu

**Affiliations:** College of Horticulture, Gansu Agriculture University, Lanzhou, 730070 People’s Republic of China

**Keywords:** *Brassica oleracea*, Variety, Nutrition characteristics, Comparative analysis

## Abstract

**Background:**

As the second largest leafy vegetable, cabbage (*Brassica oleracea* L. var. *capitata*) is grown globally, and the characteristics of the different varieties, forms, and colors of cabbage may differ. In this study, five analysis methods—variance analysis, correlation analysis, cluster analysis, principal component analysis, and comprehensive ranking—were used to evaluate the quality indices (soluble protein, soluble sugar, and nitrate), antioxidant content (vitamin C, polyphenols, and flavonoids), and mineral (K, Ca, Mg, Cu, Fe, Mn, and Zn) content of 159 varieties of four forms (green spherical, green oblate, purple spherical, and green cow heart) of cabbage.

**Results:**

The results showed that there are significant differences among different forms and varieties of cabbage. Compared to the other three forms, the purple spherical cabbage had the highest flavonoid, K, Mg, Cu, Mn, and Zn content. A scatter plot of the principal component analysis showed that the purple spherical and green cow heart cabbage varieties were distributed to the same quadrant, indicating that their quality indices and mineral contents were highly consistent, while those of the green spherical and oblate varieties were irregularly distributed. Overall, the green spherical cabbage ranked first, followed by the green cow heart, green oblate, and purple spherical varieties.

**Conclusions:**

Our results provide a theoretical basis for the cultivation and high-quality breeding of cabbage.

**Supplementary Information:**

The online version contains supplementary material available at 10.1186/s12870-024-04857-4.

## Introduction

Cabbage (*Brassica oleracea* L. var. *capitata*), which belongs to the Cruciferae family, is one of the most important vegetables grown worldwide [1]. It is a shallow-rooted cool-season crop cultivated for its large leafy head. Cabbage originated in Western Europe; it was introduced to China in the 16th century and has gradually developed into one of the country’s most important green leafy vegetables. In recent years, with the improvement of living standards and the public’s nutritional requirements, the demand for high-quality and varied vegetable products is increasing [2, 3]. Additionally, consumers not only require a steady supply of cabbage but are also increasingly concerned about its appearance, taste, and nutritional value. The different varieties of cabbage differ in mineral, nutrient, and antioxidant content. Therefore, knowing the differences between the main cabbage varieties is essential for maintaining a balanced diet [4, 5].

Studies have shown that dietary vegetables with high contents of soluble sugar, soluble protein, and vitamins reduce the risk of gastrointestinal diseases and cancer [6, 7]. Accordingly, cabbage is highly favored for its abundant soluble sugars, soluble proteins, and vitamins, as well as its high levels of bioactive non trophic compounds [8, 9]. With the development of natural phytochemicals as potential chemo preventive agents, research into the exploitation of vegetables as potential sources of natural chemo preventives has expanded. For instance, the water-soluble vitamin C (VC), flavonoids, phenols, and glucosinolates in cabbage are involved in the first and second lines of defense against oxidative stress in humans [10, 11].

Phenolic acids and flavonoids, collectively known as polyphenols, are secondary metabolites widely found in plants, including cabbage [12, 13]. Studies have shown that polyphenols have a high degree of species diversity and multiple pharmacological properties, such as anti-allergic, anti-inflammatory, antioxidant, anti-mutagenic, and anti-cancer properties [14, 15], while flavonoids act as scavengers for reactive oxygen species and electrophiles, reducing the risk of cardiovascular disease in vivo [16]. Moreover, in addition to polyphenols and flavonoids, glucosinolates are important bioactive compounds in cabbage and are also beneficial for health [17–20], and cabbage is also a rich source of essential minerals, such as iron (Fe), calcium (Ca), magnesium (Mg), potassium (K), sodium (Na), and phosphorus (P) [21].

Because of the abundance of nutrients in cabbage and its beneficial secondary metabolites, improving the nutritional quality of cabbage has become a focus of researchers. The mineral content and secondary metabolites of cabbage are affected by many factors, including environmental and cultivation conditions, as well as the variety itself. The first step of quality breeding is understanding the differences in nutritional indices among the different varieties. To meet consumers’ different requirements, breeders must cultivate several varieties of cabbage that differ in terms of shape, size, color, and leaf ball texture [22, 23]. Different varieties of vegetable typically may show differences in nutrient value, antioxidant content, and mineral content. For instance, a study of the different varieties of broccoli showed that their quality indices differ significantly [24, 25]. Meanwhile, many of the published studies on cabbage quality mainly focused on the influence of different cultivation conditions and seasons on nutritional quality and only considered a few cabbage varieties, making the results of these studies difficult to compare. Moreover, the determination methods used in these studies are also different, leading to diversification of results, and there are few comprehensive comparative analyses of the nutritional value, antioxidant content, and mineral content of different varieties and forms of cabbage cultivated under the same conditions [26].

In this study, 159 cabbage varieties in four different forms were cultivated under the same conditions in the Yuzhong area. The quality indices and antioxidant and mineral content of each were determined. Different analysis methods were used to comprehensively explore the relationships among the quality indices, nutritional value, antioxidant content, and mineral contents of the different forms of cabbage. Thus, this study’s results will provide a scientific basis for a more comprehensive evaluation of the nutritional value of cabbage and morphological differences in cabbage, as well as a theoretical basis for improving its breeding and agricultural production.

## Materials and methods

### Materials and field management

The 159 cabbage varieties in four different forms, i.e., 142 green spherical (F1), eight green oblate (F2), six purple spherical (F3), and three green cow heart (F4), were cultivated in Kangyuan Agricultural Demonstration Park, Lanzhou, Gansu Province, China (35°85’N, 104°12’E), from July–November 2022. During the cultivation period, all the varieties (Supplementary Information Table S1) were grown under the same cultivation conditions and daily management. Specifically, when the seedlings were 30 days old, they were planted at an inter-plant spacing of 30 cm and an inter-row spacing of 45 cm. During the cultivation period, fertilizer and water management was done according to local conventional methods, and mature leaf balls were collected for subsequent experiments. Each variety was harvested in the optimal harvesting period, according to the density of the leaf ball. For each variety, three disease- and pest-free plants of the same leaf-ball size and maturity were selected.

### Sample preparation

All cabbage varieties were processed on the day of harvesting. The entire leaf ball was cut and mixed after removing the rosette leaves. The mixed leaves were divided into two parts; one part was used to determine the quality and antioxidant content, while the other part was placed in an envelope and heated in an oven at 105 °C for 15 min, after which the temperature was set to 80 °C until the leaves were completely dry. The dried leaves were used to determine the mineral content of the variety. These procedures were performed in a laboratory at Gansu Agricultural University.

### Nutritional quality determination

The soluble protein content of the cabbages was determined using the Coomassie brilliant blue method. First, fresh leaves (0.5 g) were added to 5 mL of ultrapure water and rapidly ground. Then, the mixture was poured into a centrifuge tube and centrifuged at 10,000 rpm for 10 min. The supernatant was used for the determination [27].

During the determination of soluble sugar content, 0.2 g of the sample was placed in a 25 L test tube. Then, 10 mL distilled water was added, and the sample was heated to 100 °C for 1 h. Next, the liquid was filtered into a 25 mL volumetric flask, and 0.5 mL was transferred to a 20-mL test tube and mixed with 1.5 mL of ultrapure water, 0.5 mL of anthrone ethyl acetate solution, and 5 mL of concentrated sulfuric acid. The test tube was then placed in a boiling water bath for 1 min before cooling to room temperature, and the absorbance of the solution was determined at 630 nm, with a blank as reference [28].

The nitrate content of the samples was determined using the salicylic acid method [29]. First, 3.0 g of fresh cabbage leaves was weighed and transferred into a test tube containing 10 mL of ultrapure water and placed in a boiling water bath for 30 min. Afterwards, the mixture was cooled to room temperature and filtered into a 25 mL volumetric flask, and the volume was adjusted to 25 mL using ultrapure water. Next, 0.1 mL of the filtered supernatant was transferred to a test tube, and 0.4 mL of 5% salicylic acid/sulfuric acid solution was added. Finally, 9.5 mL of 8% NaOH solution was added slowly, and the absorbance of the solution was measured at 410 nm.

### Antioxidant indices determination

To determine the VC content, 0.5 g of the sample was grounded, and 1.5 mL of 2% oxalic acid was added. Then, the sample was mixed with 0.5 mL of 30% zinc sulfate and 0.5 mL of 15% potassium ferrocyanide. Then, the VC content was determined using the 2,6-dichlorophenolate method [30].

To determine the glucosinolate content, 0.1 g of the sample was placed in a 10 mL test tube and set in a boiling water bath for 10 min. After adding 8 mL of boiling distilled water, the sample was set in the boiling water again for 10 min. The volume of water was increased to 10 mL and cooled to room temperature, and the sample was filtered. Then, 2 mL of the filtrate was transferred into a 10 mL cuvette tube, and 4 mL of 0.15% sodium carboxymethyl cellulose was added. After shaking at 22 ± 3 °C for 2 h, 2 mL of 8-mmol/L palladium chloride color development solution was added, and the absorbance of the solution was measured at 540 nm [31].

Next, 0.2 g of the sample was homogenized with 6 mL of 80% ethanol. Then, the ethanol extract was centrifuged at 12,000 rpm and 4 °C for 20 min, and the total phenols and flavonoids were determined using the resulting supernatant [32]; the absorbance of the solution was determined at 765 nm (with Folin-Ciocalteu colorimetry) [33] and 510 nm (with aluminum chloride colorimetry) [34].

### Determination of mineral content

First, 0.5 g of the dried sample was dissolved in 5 mL of concentrated sulfuric acid for 12 h. When the solution was completely black, the triangular was placed on an electric furnace and heated. After a large amount of white smoke was produced, 5–10 drops of 30% H_2_O_2_ were added, and this procedure was repeated 3–5 times. Each time, the amount of H_2_O_2_ added was decreased, and the solution was boiled until it became clear. Then, the triangular was cooled. The boiled solution was filtered into a 50 mL reagent bottle for determination. The K, Ca, Mg, Cu, Fe, Mn, and Zn content of the solution were determined by atomic flame absorption spectrophotometry (ZEEnit700p, Germany) [35].

### Data analysis

The average values (means) and standard errors (SEs) of the nutritional value, mineral content, and antioxidant content were calculated using Microsoft Excel 2013 (three replications were used to calculate the average values). SPSS Version 23.0 software (IBM Corp., Armonk, NY, United States) was used for one-way analysis of variance and Pearson correlation analysis. Significant difference levels were set at *p* < 0.05 and *p* < 0.01. Principal components analysis (PCA) and cluster analysis were performed using SPSS and Origin Pro 2018 (Origin-lab Corporation, Northampton, MA, USA), respectively.

PCA was done using the idea of dimensional reduction, a multi-index that can be transformed into several statistical methods of comprehensive index. Firstly, the average value of each sample index was inputted into SPSS Version 23.0 as a variable. Then, the coefficient (load value) for important components and their eigenvalues were used to calculate the eigenvectors (Table 1). Using the eigenvectors as weighting factors, the functional expressions for each principal component are:

**Table 1:** Principal component load matrix and eigenvector for each substance

Index	Load value			Eigenvector		
	PC1	PC2	PC3	PC1	PC2	PC3
Soluble protein	0.631	−0.757	0.171	0.251	−0.322	0.116
Soluble sugar	−0.873	0.447	0.195	−0.348	0.19	0.133
Nitrate	0.986	0.075	−0.146	0.393	0.032	−0.099
VC	−0.694	−0.716	−0.077	−0.276	−0.304	−0.052
Glucosinolates	−0.871	0.206	−0.446	−0.347	0.088	−0.304
Polyphenol	−0.76	0.003	0.65	−0.303	0.001	0.443
Flavonoid	0.8	0.455	−0.391	0.319	0.193	−0.266
K	−0.02	0.996	0.084	−0.008	0.423	0.057
Ca	−0.271	0.714	0.645	−0.108	0.303	0.439
Mg	0.518	0.414	0.749	0.206	0.176	0.51
Cu	−0.064	0.94	−0.335	−0.025	0.399	−0.228
Fe	0.115	−0.929	0.353	0.046	−0.395	0.24
Mn	0.67	0.73	0.139	0.267	0.31	0.095
Zn	0.952	−0.218	0.216	0.379	−0.093	0.147

$$\begin{aligned} {Y_1} & =0.251{X_1} - 0.348{X_2}+0.393{X_3} \\ & \quad - 0.276{X_4} - 0.347{X_5} - 0.303{X_6} \\ & \quad +0.319{X_7} - 0.008{X_8} - 0.108{X_9} \\ & \quad +0.206{X_{10}} - 0.025{X_{11}}+0.046{X_{12}} \\ & \quad +0.267{X_{13}}+0.379{X_{14}} \\ \end{aligned}$$

$$\begin{aligned} {Y_2} & = - 0.322{X_1}+0.19{X_2}+0.032{X_3} \\ & \quad - 0.304{X_4}+0.088{X_5}+0.001{X_6} \\ & \quad +0.193{X_7}+0.423{X_8}+0.303{X_9} \\ & \quad +0.176{X_{10}}+0.399{X_{11}} - 0.395{X_{12}} \\ & \quad +0.31{X_{13}} - 0.093{X_{14}} \\ \end{aligned}$$

$$\begin{aligned} {Y_3} & =0.116{X_1}+0.133{X_2} - 0.099{X_3} \\ & \quad - 0.052{X_4} - 0.304{X_5}+0.443{X_6} \\ & \quad - 0.266{X_7}+0.057{X_8}+0.439{X_9} \\ & \quad +0.51{X_{10}} - 0.228{X_{11}}+0.24{X_{12}} \\ & \quad +0.095{X_{13}}+0.147{X_{14}} \\ \end{aligned}$$

The principal component synthesis model was calculated using the ratio of the corresponding eigenvalues of the three principal components and the extracted total eigenvalues of the principal components as weights:

$$Y=0.45{Y_1}+0.396{Y_2}+0.154{Y_3}$$

## Results

### Nutritional indices and antioxidant and mineral content of the different varieties of cabbage

**Fig. 1:**
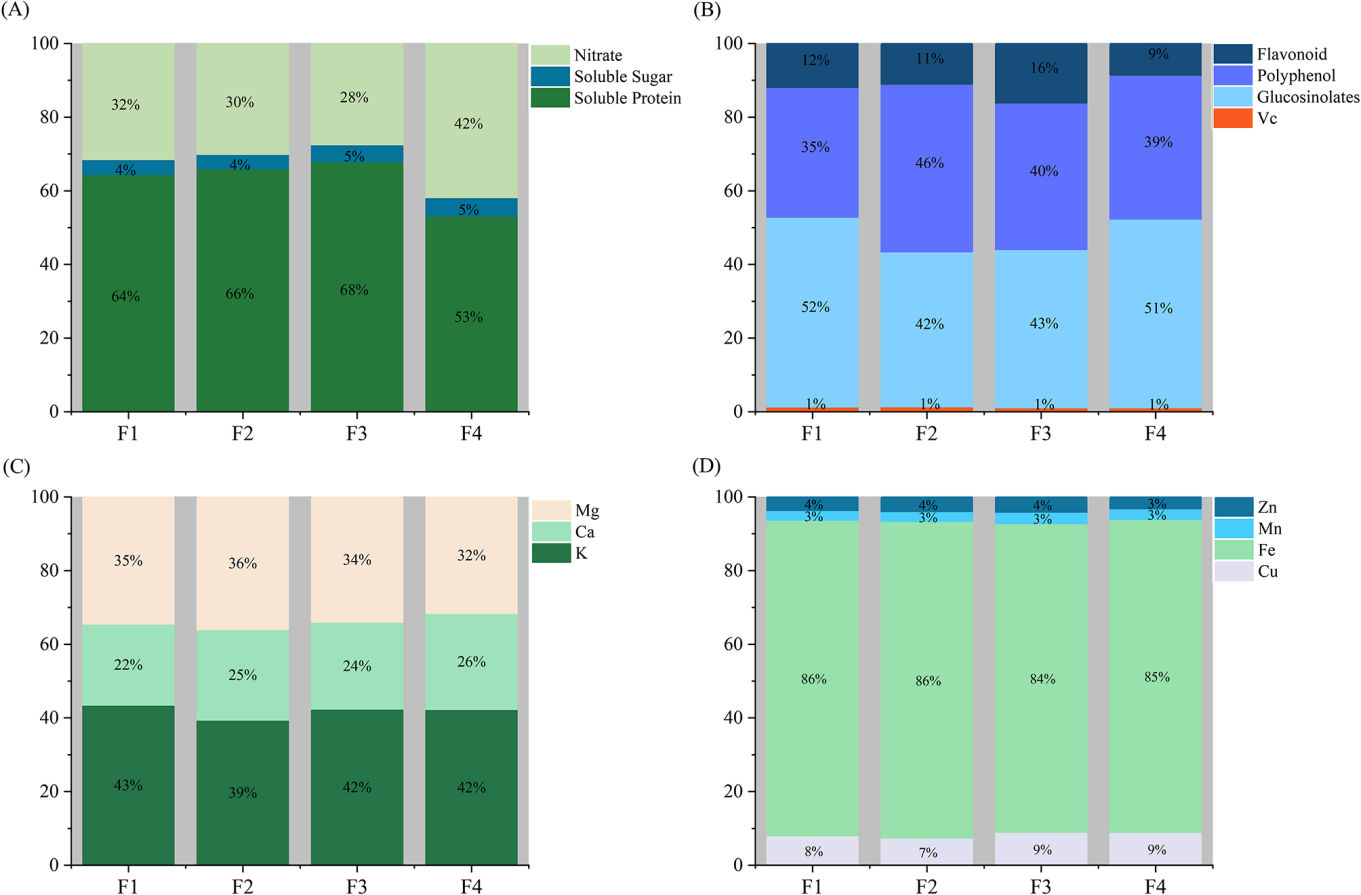
Stacking bar plots showing the quality indices and antioxidant and mineral contents of the four cabbage forms. F1 (green spherical form), F2 (green oblate form), F3 (purple spherical form), and F4 (green cow heart form)

Our results show that there are significant differences in the nutrient, antioxidant, and mineral content of the 159 different varieties of cabbage (Supplementary Information Table S2). From the main related quality indices shown in Table 2, the soluble sugar content of the green oblate form is highest. The green cow heart form has the highest soluble protein content, significantly higher than those of the other three forms. Regarding the proportion of nutrients in the different cabbage forms, the purple spherical form had the highest nutrient quality, with the lowest nitrate content accounting for 28% of the total, the highest soluble protein content accounting for 68%, and the sugar content accounting for 5% (Fig. 1A). Since a higher nitrate content has a greater impact on human health, and soluble protein and sugar are important vegetable quality indices, we can conclude that the purple spherical cabbage has a quality advantage.

**Table 2:** Mean ± standard error (SE), range, and coefficient of variation (CV) for the quality trait species analyzed in the collection

Traits	F1 (green spherical form)			F2 (green oblate form)			F3 (purple spherical form)			F4 (green cow heart form)		
	Mean ± SE	Range	CV (%)	Mean ± SE	Range	CV (%)	Mean ± SE	Range	CV (%)	Mean ± SE	Range	CV (%)
Soluble protein (mg/kg)	381.1 ± 8.12 a	189.4–756.3	25.4	410.5 ± 36.11 a	236.3–558.4	24.9	357.8 ± 7.03 a	172–368.3	4.8	296.2 ± 19.08 a	260.5–402.5	11.2
Soluble sugar (mg/kg)	23.96 ± 0.38 a	13.2–36.5	18.9	24.28 ± 1.38 a	19–30.5	16.1	23.97 ± 1.16 a	19.8–27.2	11.8	27.27 ± 3.99 a	22.5–35.2	25.4
Nitrate (mg/kg)	186.7 ± 3.9 ab	105.1–284.7	24.9	187.9 ± 20.6 ab	118.2–266.6	31	145.4 ± 6.03 b	116.1–254.4	10.2	233.3 ± 14.6 a	154.7–239.9	10.8
Vc (mg/g)	0.32 ± 0.01 a	0.184–0.686	31.3	0.31 ± 0.04 a	0.164–0.528	35.5	0.25 ± 0.01 a	0.202–0.284	13.3	0.31 ± 0.02 a	0.285–0.345	10.8
Glucosinolate (umol/g)	13.2 ± 0.38 a	3.8–27.87	34.3	9.98 ± 0.95 a	6.01–13.36	26.9	10.54 ± 0.75 a	6.85–16.87	17.5	15.05 ± 0.49 a	9.08–15.61	5.6
Polyphenol (mg/g)	9.02 ± 0.32 a	3.1–18.48	42.5	10.8 ± 1.9 a	4.34–17.28	49.6	9.75 ± 0.97 a	3.48–15.85	27.4	11.48 ± 0.94 a	8.85–13.33	14.2
Flavonoid (mg/g)	3.08 ± 0.12 a	1.27–8.77	46.8	2.64 ± 0.18 a	1.85–3.32	18.9	3.98 ± 0.24 a	2.58–4.76	15.1	2.56 ± 0.31 a	1.96–2.96	20.7
K (mg/kg)	8076.4 ± 85.49 a	5860–10,540	12.6	8233.7 ± 334.4 a	6893–9833	11.5	9511.6 ± 409.3 a	8446–11,120	10.5	9372.2 ± 720.1 a	8583–10,810	13.3
Ca (mg/kg)	4078.6 ± 63.08 b	2337.3–5635	18.4	5143.6 ± 213.8 ab	3878.–5790.	11.8	5293.8 ± 189.8 a	4417–5700	8.8	5777.2 ± 203.4 a	5378–6045	6.1
Mg (mg/kg)	6433.3 ± 119.5 a	2516–10,060	22.1	7549.1 ± 502.5 a	5586–9606	18.8	7656.6 ± 509.7 a	6496.7–9973	16.3	7031 ± 1013.9 a	5003.3–8050	25
Cu (mg/kg)	6.03 ± 0.14 a	2.73–11.21	27.7	5.64 ± 0.5 a	3.12–7.44	25	6.64 ± 1.12a	3–10.71	41.3	6.54 ± 0.54 a	5.57–7.44	14.4
Fe (mg/kg)	64.74 ± 0.56 a	50.68–90.42	10.3	66.29 ± 2.85 a	58.27–78.23	12.2	62.61 ± 1.48 a	59.13–68.78	5.8	62.75 ± 3.85 a	55.63–68.85	10.6
Mn (mg/kg)	1.99 ± 0.02 a	1.37–2.57	12.6	2.06 ± 0.05 a	1.86–2.28	7.3	2.31 ± 0.07 a	2.1–2.59	7.4	2.06 ± 0.02 a	2.03–2.09	1.5
Zn (mg/kg)	2.84 ± 0.08 a	0.46–5.57	31.7	3.11 ± 0.28 a	1.6–3.89	25.4	3.15 ± 0.61 a	0.97–4.71	47.3	2.45 ± 0.6 a	1.25–3.13	42.4

Different alphabet represents significant differences (*p* < 0.05) (Tukey test)

Regarding antioxidant indices (Table 2), the glucosinolate and polyphenol contents of the green cow heart form were the highest at 15.05 mg/g and 11.48 mg/g, respectively. The glucosinolate content of the green oblate form and the polyphenol content of the purple spherical form were the lowest at 9.98 mg/g and 8.75 mg/g, respectively. The VC content of the green spherical form was the highest, 28% higher than that of the purple spherical form, and there was no significant difference in VC content between the green spherical and green oblate forms.

Regarding antioxidant content (Fig. 1B), the highest proportion of glucosinolates in the green spherical form and polyphenols in the green oblate form were 52% and 46%, respectively. The proportion of flavonoids in the purple spherical form was the highest at 16%, which was 8% higher than that of the green cow heart form. As shown in Fig. 1B, among the four antioxidant indices, glucosinolate accounted for the highest proportion (accounting for more than 40%) of the same form, followed by polyphenols, flavonoids, and VC.

Regarding mineral composition, the K, Mg, Cu, Mn, and Zn content of the purple spherical form were the highest, reaching 9,511.6 mg/kg, 7,656.6 mg/kg, 6.64 mg/kg, 2.31 mg/kg, and 3.15 mg/kg, respectively. Moreover, the Fe content of the green oblate form (66.29 mg/g) and the Ca content of the green cow heart form (5,777.2 mg/kg) were the highest, and there was no significant difference in Fe content among the other three forms. The proportion of each element was different in the four forms of cabbage, resulting in different richness (Fig. 1C and D). K and Fe accounted for more than 39% and 80% of the total macroelement and trace element content of the four cabbage forms, respectively. Fe was the most abundant trace element (F1 86%, F2 86%, F3 84%, and F4 85%), while K was the most abundant macroelement (F1 43%, F2 39%, F3 42%, and F4 42%).

There are significant differences in the nutrient, antioxidant, and mineral contents of the four cabbage forms (Table 2). Specifically, several indices show coefficients of variation (CVs) higher than 40%. The flavonoid CV of the green spherical form was 46.8%, the polyphenol CV of the green oblate form was 49.6%; the Cu CV of the purple spherical form was 41.3%, and the Zn CV of the green cow heart form was 42.4%. The degrees of variation of these four indices are significantly higher than those of other indices for the same morphology. Conversely, the Fe CV of the green spherical form was 10.3%, and the Mn CV of the green oblate and cow heart forms were the lowest at 7.3% and 1.5%, respectively. Furthermore, the soluble protein CV of the purple spherical form was the lowest at 4.8%, indicating that the Fe content of the green spherical form, the Mn content of the green oblate spherical and cow heart forms, and the soluble protein content of the purple spherical form have the highest regularity among the four forms of cabbage studied.

### Correlation analysis

**Fig. 2:**
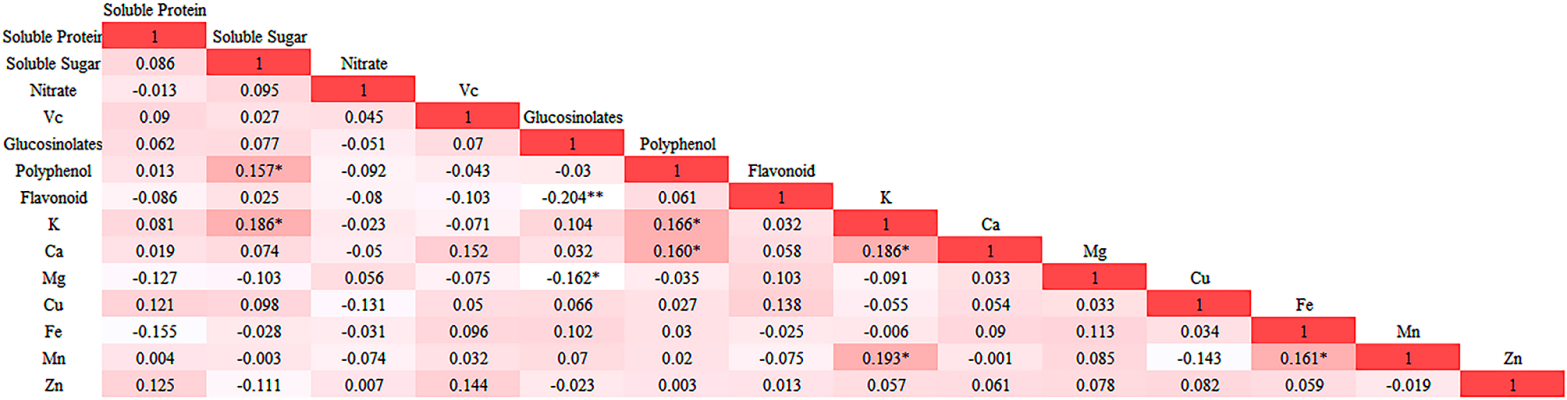
Correlation matrix based on Pearson correlation coefficient of the different quality indices and antioxidant and mineral content. The colors and values represent the degree of correlation proportional to the correlation coefficient; a deeper color represents greater correlation. *, ** Represents significance at *p* < 0.05 and *p* < 0.01, respectively

Pearson correlation analysis (PCA) can reveal the correlations between two parameters. We found multiple sets of significant correlations among the 14 indices (Fig. 2). Soluble sugar was significantly positively correlated with polyphenol and K (*p* < 0.05); glucosinolate was significantly negatively correlated with Mg and negatively with flavonoid, and polyphenol was significantly positively correlated with K and Ca (*p* < 0.05). Moreover, K was significantly positively correlated with Ca and Mn (*p* < 0.05). These results show that there are internal relationships among these indices, especially among the minerals, resulting in information overlap. Further, it was found that PCA can be performed based on the internal relationships between the correlation analysis indices.

### Heatmap visualization and hierarchical cluster analysis

**Fig. 3:**
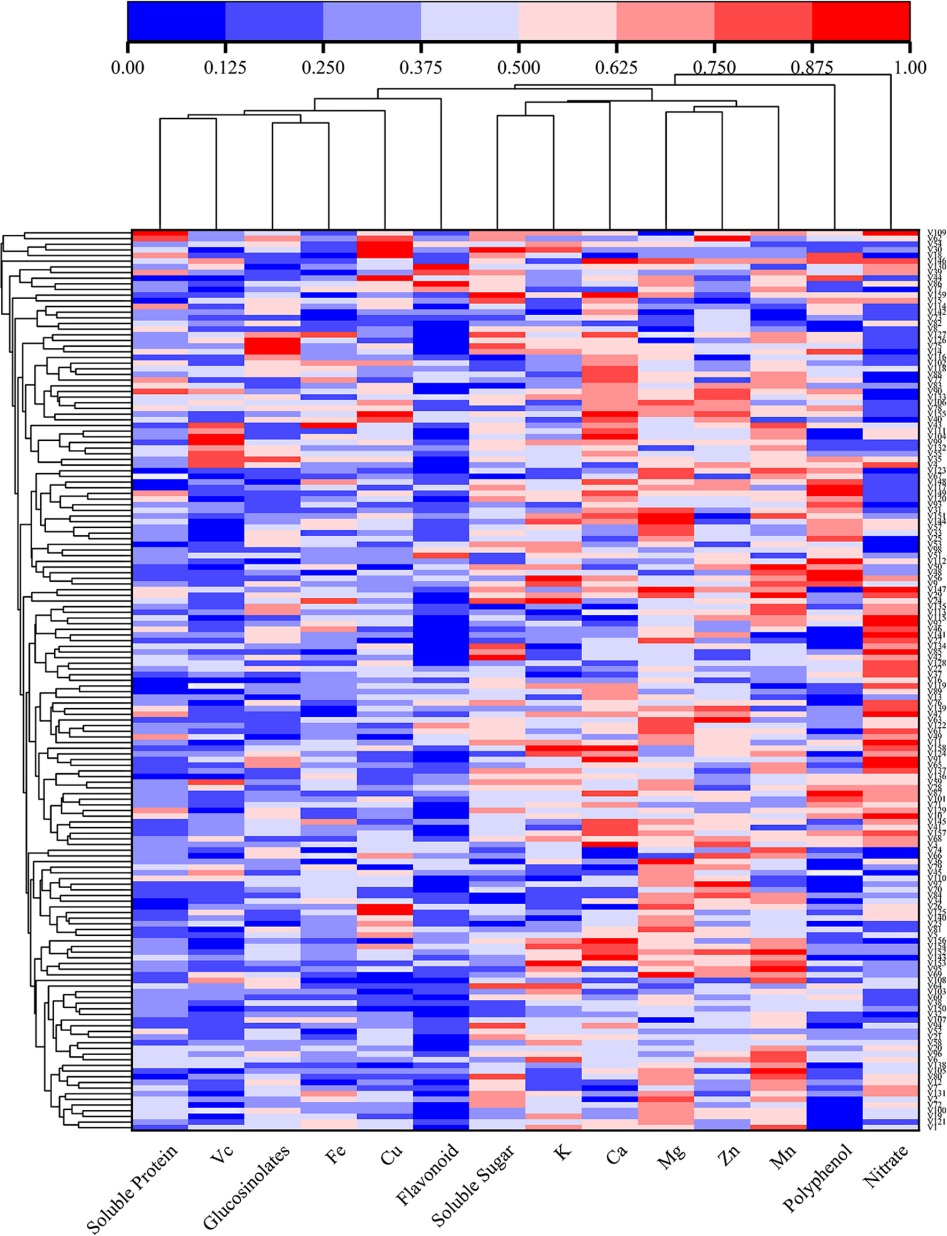
Hierarchical clustering and heat map visualization of the quality indices and antioxidant and mineral contents of the 159 cabbage varieties. V1–V159 represent 159 varieties. The red, blue, and white indicate high, low, and medium content, respectively

Cluster analysis is a method widely used to summarize and aggregate complex multidimensional and group data attributes with the same characteristics. In this study, the nutrient, antioxidant, and mineral contents of the 159 varieties of cabbage were determined by heatmap analysis. All data were normalized between 0 and 1 for use on the generated heatmap based on Pearson correlation coefficients. The changes in the measured indices are illustrated in the form of heatmap clustering in Fig. 3. The clustering divided the indices into two categories. The most unique category was nitrate, excessive contents of which can cause harm. Cluster analysis classified it as a separate category, clearly discriminating between harmful and beneficial substances. In addition, the colors for K, Ca, Mg, Zn, Mn, soluble sugar, nitrate, and polyphenol in Fig. 3 are closer to red than those for soluble protein, VC, glucosinolate, flavonoid, Cu, and Fe, indicating that their contents are higher, as well as demonstrating the utility of heatmap visualization. In this study, there were many varieties and rich clustering levels. When the classification level is lower, more similar varieties are clustered into the same class, such as V152, V153, V154, and V156, and it is worth noting that these varieties were all purple spherical cabbages, indicating that these varieties have high similarity.

### Principal component scatter plots and comprehensive evaluation

#### Scatter plots of principal components for the different varieties

**Fig. 4:**
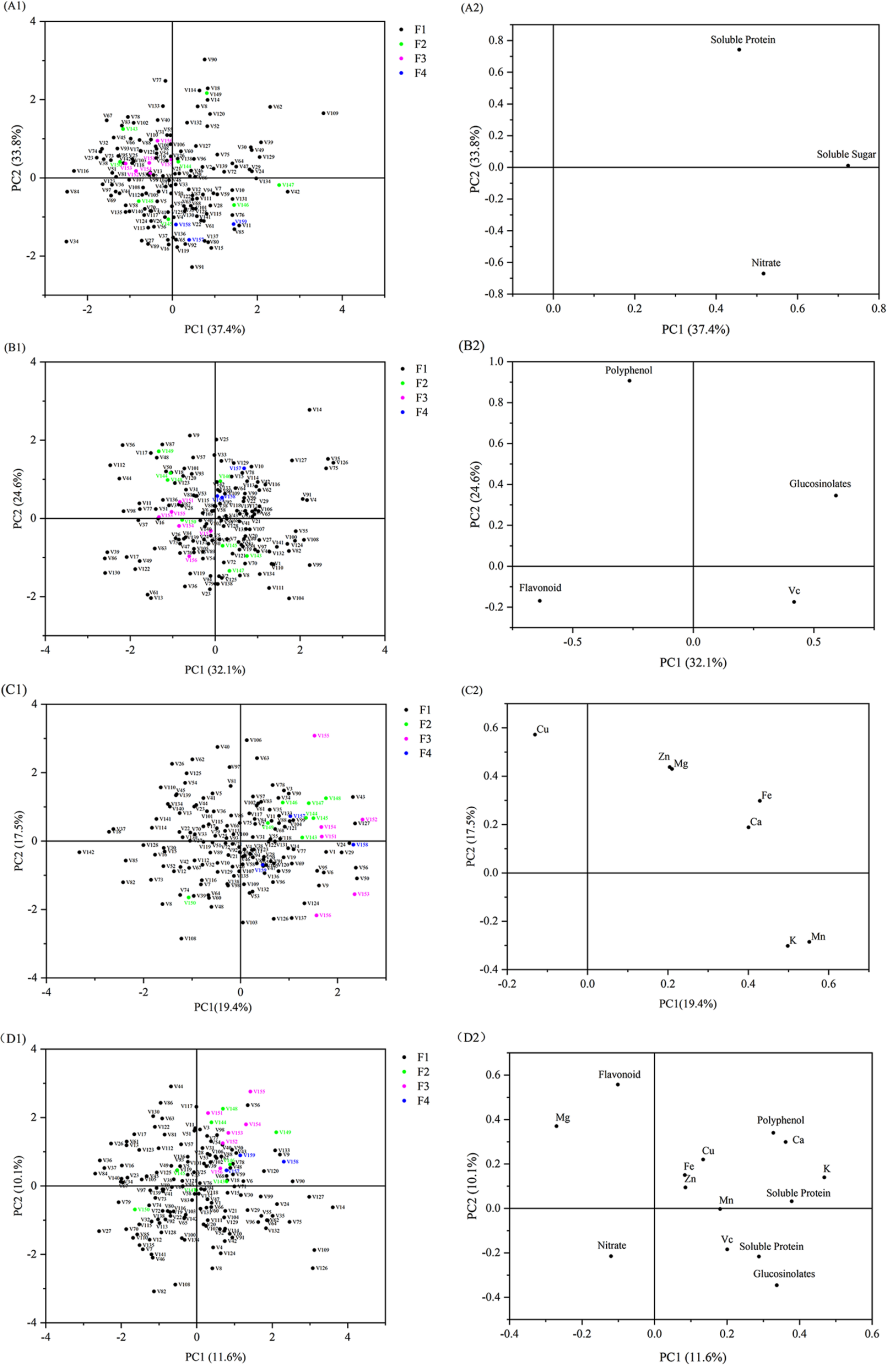
Principal component analysis (PCA) of 159 cabbage varieties and 14 indices. (**A1**–**D1**) PCA scatter plot showing varieties; (**A2**–**D2**) PCA loading diagram showing indices

Figure 4A1, A2 show the scatter plots of nutritional value. The two principal components PC1 and PC2 accounted for 37.4% and 33.8% of the total variance (i.e., 71.2% in total), respectively. From Fig. 4A1, it can be seen that the six purple spherical varieties are all distributed to the second quadrant, and the three green cow heart varieties are all in the fourth quadrant, indicating a high level of similarity between them, while the green spherical and oblate forms are distributed over the four quadrants, indicating that they have obvious differences. From a distribution perspective, the purple spherical form is dominated by the value of the second principal component, and the green cow heart form is dominated by the first principal component. From Fig. 4A2, we can conclude that the main index of the first principal component is soluble sugar, while that of the second principal component is soluble protein.

From the scatter plots of antioxidant content (Fig. 4B1, B2), it can be seen that the green spherical and green oblate forms are widely distributed over four quadrants. The purple spherical form is distributed to the second and third quadrants in the antioxidant PCA plot, indicating that there are some differences in its antioxidant indices, while the three green cow heart varieties V157, V158, and V159 are distributed to the first quadrant, indicating that their antioxidant capacity similarity is high. Glucosinolate is the main contributor to the first principal component, and polyphenol is the main contributor to the second principal component (Fig. 4B2). The glucosinolate and polyphenol values of V157, V158, and V159 are higher than those of the other varieties, which is reflected in their distribution to the first quadrant.

Figure 4C1, C2 show the PCA plots of mineral content. In Fig. 4C1, the purple spherical and green cow heart varieties are distributed to the first and fourth quadrants. From Fig. 4C2, it can be seen that the three most important indices contributing to the first principal component are Mn, K, and Fe. Thus, the distributions of the purple spherical and green cabbage forms are determined by these three indices.

By analyzing the nutritional quality, antioxidant content, and mineral content of these varieties, we were able to summarize and analyze the PCA diagrams of 14 indices (Fig. 4D1, D2). The purple spherical and green cow heart forms were still densely distributed, unlike the other two forms (Fig. 4D1). The diagrams in Fig. 4A1–C1 show the same results. Therefore, it can be concluded that the purple spherical and green cow heart forms have certain quality stability advantages over the other two forms.

Through the scatter plots, we confirmed the main nutrients, antioxidants, and minerals present in the 159 cabbage varieties and evaluated the differences in the components of the different varieties, which is helpful for comprehensively investigating the primary and secondary metabolites in cabbage.

#### Comprehensive evaluation of the principal components of the different cabbage forms

PCA was performed on 14 indices for the four forms (Table 3). Three principal components were extracted, and the cumulative variance contribution reached 100%, explaining all the raw information. It can be seen in Tables 1 and 3 that the first principal component accounts for 45.0% of the variance and is mainly characterized by high load values for soluble protein, nitrate, flavonoid, and Zn. The variance contribution rate of the second principal component is 39.6%, and the load values of soluble sugar, glucosinolates, K, Ca, Cu, and Mn are high, being the main indices contributing to the second principal component. The variance contribution rate of the third principal component is 15.4%, and the load values of the principal components VC, polyphenol, Mg, and Fe are high, being the main indices contributing to the third principal component.

**Table 3:** Factor analysis for each component

Component number	Eigen value	Variance contribution (%)	Cumulative variance contribution (%)
Principal component 1	6.30	45.02	45.02
Principal component 2	5.54	39.58	84.60
Principal component 3	2.16	15.4	100

The final scores are shown in Table 4. The purple spherical form scored the highest, indicating that the nitrate content of the purple spherical form is low, and its flavonoid, soluble protein, and Zn contents are high because a comprehensive evaluation of PC1 showed that they accounted for 45.0% of the total contribution rate, representing most of the information for the 14 indices. The scores for the other forms decreased in the following order: green cow heart > green oblate > green spherical. Following a comprehensive ranking of the principal components of the 159 varieties (Supplementary Information S3), the top 10% (16 varieties) were V151, V144, V159, V155, V157, V152, V158, V146, V156, V143, V154, V147, V106, V145, V31, and V153. Thus, the score for the green spherical form was lower than that for the other three forms. A comprehensive data analysis of the principal component ranking for the varieties revealed results which were consistent with those of the morphological principal component ranking.

**Table 4:** Comprehensive scores for the four forms

Form	Y_1_	Y_2_	Y_3_	Y	Rank
F1 (green spherical form)	8.32	56.39	56.12	34.72	4
F2 (green oblate form)	9.53	62.14	66.63	39.16	3
F3 (purple spherical form)	9.53	68.40	68.44	41.91	1
F4 (green cow heart form)	7.19	68.35	67.31	40.67	2

The analysis methods and formulas for variety ranking (Supplementary Information Table S3) are the same as those in Table 4. The principle coefficient of important components (load value) and the eigenvalues of the principal components were used to calculate the feature vector (Supplementary Information Table S4). Taking the feature vector as the weight, the function expression for each principal component was constructed. The seven principal components (Supplementary Information Table S5) and the proportion of the corresponding eigenvalues of each principal component to the total eigenvalues of the extracted principal component were used as weights to calculate the principal component synthesis model.

## Discussion

This study provides a comprehensive comparison of the nutritional quality, antioxidant content, and mineral content of different varieties of cabbage. Many varieties of cabbage were cultivated under the same environmental conditions. In previous studies, differences in the morphologies and varieties of cabbage during domestication and evolution were identified [36]. In this study, 159 varieties of four different forms were compared and distinguished, revealing that the shape, color, and mineral contents of the different forms and varieties were significantly different and fully confirming their diversity and differences.

### Differences in nutrient contents among the different forms of cabbage

Green cabbage is the most common cabbage form, but purple cabbage has more nutritional value, and previous studies have indicated the health benefits of consuming more chemoprotective substances [37]. In this study, by comparing 14 indices of cabbage, the comprehensive quality of the purple spherical cabbage was found to be higher than that of the green spherical cabbage. Studies on pumpkin and mulberry have shown that the carotenoid contents of different pumpkin varieties and the active ingredients and antioxidant activities of different varieties of young mulberry leaves are significantly different [38, 39]. In the current study, we also found significant differences among the different forms of cabbage. The nitrate content of purple cabbage was the lowest; the soluble protein content of the green oblate cabbage was the highest, and the soluble sugar content of the cow heart cabbage was the highest. This shows that breeding and cultivation selection can be performed according to the index of the variety, and this study also provides some theoretical support for that. In addition to exploring the soluble sugars and proteins present in the different cabbage forms, which are beneficial to human health, nitrate, which is an uncertain factor, was evaluated. Nitrate itself is considered relatively non-toxic, but when it accumulates to a certain extent and is absorbed, it can be converted to toxic nitrite in the human body [40, 41]. Therefore, excessive nitrate content is considered an unhealthy trait [42, 43]. Leafy vegetables are the main source of human dietary nitrate, accounting for 40–92% of the average daily intake and 70–94% of total nitrate intake [44, 45], which indicates that most of the nitrate in the body is gotten through the consumption of leafy vegetables. The 159 varieties and four forms of cabbage evaluated in this study showed significant differences in nitrate content. Consistent with the results of Czech and Rusinek, purple cabbage showed the lowest nitrate content in our study, indicating that purple cabbage varieties are superior in terms of health requirements [46].

### Differences in antioxidant contents among the different forms of cabbage

Cabbage contains various natural antioxidants, such as phenolic compounds and glucosinolates [47, 48]. For consumption and cultivation, it is important to choose vegetables with a large amount of phenol and glucosinolate compounds because they are directly related to human health [49, 50]. In particular, the proportion of glucosinolate in cooked food depends on its type and variety [51]. This is consistent with the conclusion of our study, which is that the antioxidant content of cabbage depends on the variety of the cabbage. We also found that the total polyphenol and flavonoid contents of purple cabbage were higher than those of green spherical and green oblate cabbage and lower than those of green cow heart cabbage, supporting the conclusions of Heo and Lee [52]. Therefore, the purple spherical and cow heart cabbages can be exploited as varieties rich in antioxidants. In addition to phenolics and glucosinolates, VC is a natural antioxidant in cabbage, but its contribution to the total antioxidant capacity is estimated to be less than 15% [53, 54]. In this study, we found significant differences in antioxidant content among the different forms and that the contribution of VC to the total antioxidant contents is very small (around ~ 1% for all four forms), which is consistent with previous research findings [53].

### Differences in mineral content among the different forms of cabbage

Cabbage is rich in mineral elements. Czech et al. identified several differences in the mineral content of different cabbage varieties, with the Mn content of purple cabbage being significantly higher than that of green cabbage. Other elements such as Fe, Ca, Cu, and Zn are also higher in purple cabbage than in green cabbage [46, 55]. Our study showed that the Fe and Ca contents of the purple cabbage varieties were lower than those of the green varieties; our results were different from those of previous studies probably because the varieties and planting conditions used in this study were different. In the present study, the Mn, Cu, and Zn contents of purple cabbage were found to be higher than those of green cabbage, which is consistent with reports by Czech et al. Several studies have also shown that while the accumulation of mineral elements in different varieties of cabbage is relatively uniform [56, 57], the elemental contents of cabbages planted in different regions are different due to differences in soil composition and mineral effectiveness [58].

### Correlation between the nutrients, antioxidants, and mineral content of the different forms of cabbage

Karl Pearson proposed the Pearson correlation coefficient (*R*), which is still used today [59]. *R* is an indicator of the strength of a linear correlation between two variables that takes values ranging from − 1 to 1. The closer the value of *R* is to 1, the stronger the positive correlation between the two variables is; the closer the value is to –1, the stronger the negative correlation is, and a *R* value close to or equal to 0 indicates a weak or nonlinear relationship. In this study, correlation analysis was performed on 14 indices, and multiple sets of significant or extremely significant correlations were identified, reflecting the results reported by Ning et al. that there is a correlation between polyphenols and flavonoids [60]. This indicates that correlation analysis has research significance.

A previous study on the VC, flavonoids, and nitrate contents of 27 spinach varieties found nitrate levels to be negatively correlated with the VC and total flavonoid contents [61]. Our results also showed a negative correlation between nitrate and glucosinolate, polyphenol, and flavonoid antioxidants. However, a difference is that our study showed a weak positive correlation between VC and nitrate. This is because low VC contents were observed in our study, and the difference in VC content between the varieties was not significant.

Wang et al. reported that the Ca, Fe, and Zn contents of cabbage are positively correlated [62], and our correlation analysis led to the same conclusion. Furthermore, our correlation analysis showed that the correlations among K, Mg, Cu, and Mn are different in different forms of cabbage. Several studies have also shown that the correlation between mineral elements varies in cabbage, but no relationship law has been established [63, 64].

### Cluster analysis of the different forms of nutrients, antioxidants, and mineral elements

Multivariate statistical techniques, such as multiple linear regression analysis, hierarchical cluster analysis, PCA, and factor analysis, are important tools for metabolite analysis [65]. Cluster analysis is a method widely used for summarizing complex multidimensional data, finding data item groups with the same attributes, and generating visual results [66]. For instance, a cluster analysis of the nutritional components and mineral elements of different blueberry varieties provided a scientific guide for their evaluation, consumption, and parent selection [67]. The genetic variation and diversity of certain agronomic traits and nutritional qualities of upland rice genotypes has also been analyzed by clustering methods [68]. In the course of this study, we used cluster analysis to reveal the relationships and differences among cabbage varieties and their quality indices, demonstrating that purple cabbage varieties exhibit high uniformity and varietal similarity and that the differences between the three green cabbage forms is not significant. Meanwhile, there is a significant difference between the attributes of the green and purple cabbage varieties.

### Comprehensive principal component evaluation of the nutrients, antioxidants, and mineral elements of the different forms of cabbage

PCA is a method of filtering important variables by passing multiple variables through linear transformation [69]. Using the idea of dimensional reduction, a multi-index can be transformed into several statistical methods of comprehensive index. These composite indicators retain most of the information about the original indicators and are not related to each other [70, 71]. Based on morphological and physiological data, yield and quality, performance stability, heterosis, and combination ability, Evgenidis et al. evaluated the breeding values of different tomato varieties, using PCA to comprehensively evaluate the differences between the varieties [72]. Our research process was similar to theirs. Through PCA of different varieties of cabbage, the main contribution indicators of the three principal components and the distribution and similarity among varieties were visualized in scatter diagrams. Finally, through principal component sorting, under the premise of not losing or losing little original information, the original number of related indices was converted into new independent or unrelated comprehensive indices, clearly showing the comprehensive score between the four forms (Table 4).

## Conclusions

In this paper, the main nutritional qualities, antioxidant indices, and mineral contents of four forms of cabbage cultivated under the same conditions were explored. After determining, comparing, and analyzing these indices, the overall value of purple spherical cabbage was found to be higher than that of the other three forms, but there are certain advantages among the different varieties and forms in terms of single factor contents. Our results clearly demonstrate the diversity among different forms of cabbage varieties and provide information for health-conscious consumers seeking balanced diets based on the nutritional and functional characteristics of different cabbage varieties and forms. Furthermore, our study provides a scientific basis for the selection and quality breeding of cabbage.

### Electronic supplementary material

Below is the link to the electronic supplementary material.

Supplementary Material 1

Supplementary Material 2

Supplementary Material 3

Supplementary Material 4

Supplementary Material 5

Supplementary Material 6

## Data Availability

All data generated or analysed during this study are included in this published article (and its supplementary information Table S6 files). We hereby declare that the cabbage material information used in this study is included in Supplementary Information Table S1 and have the right to use them.
